# The Buffalo Resolutions

**Published:** 1896-09

**Authors:** 


### THE BUFFALO RESOLUTIONS.

*Whereas*, His honor, acting Mayor Boeckel, with all the numerous demands which have been made upon his time, has honored us by opening our Convention and extending a cordial welcome and the freedom of the city; be it

*Resolved*, That we, the members of the United States Veterinary Medical Association, recognizing these facts, and with a full appreciation of the same, extend to him a hearty vote of thanks. (Carried unanimously.)

---

*Whereas*, The thirty-third annual meeting of the United States Veterinary Medical Association in Buffalo has been so enjoyable and profitable as to mark the beginning of a new era in the history of our organization; and,

*Whereas*, This gratifying state of affairs has resulted largely from the efforts of the energetic and hospitable local committees; be it

*Resolved*, That this Association tender to the local committees and those who have assisted them a vote of thanks, to indicate its sincere appreciation of the reception it has been accorded in Buffalo, and recommends that the President appoint a committee to draw up suitable resolutions expressive of our appreciation, and that the same be engrossed in permanent form and presented to the Chairman of the Local Committee of Arrangements, and that a copy of the same be spread upon the minutes of this meeting.

---

*Resolved*, That the United States Veterinary Medical Association, assembled in annual convention at Buffalo, N. Y., September 3, 1896, hereby respectfully and most earnestly protests against the passage, by the Congress of the United States, of Senate Bill No. 1552, improperly entitled "A Bill for the Further Prevention of Cruelty to Animals in the District of Columbia," or of any other bill designed like the said bill, to place restrictions upon scientific experiments upon living animals.

*Resolved*, That the said Association believes such legislation to be injurious to the progress of the biological sciences, and that where operative it will prevent the acquirement of medical knowledge essential for the prevention and cure of the diseases of animals and men, and desires to add its protest to the protests already made by the American Medical Association, the National Academy of Sciences, the Association of American Medical Colleges, the Association of American Physicians, the American Academy of Medicine, the Medical Society of the District of Columbia, the American Association for the Advancement of Science, the American Microscopical Society, and other medical and scientific societies of the United States.

*Resolved*, That the Secretary be directed to transmit a copy of these resolutions to the President of the Senate and the Speaker of the House of Representatives.

---

*Resolved*, That the United States Veterinary Medical Association appreciates the opportunity so courteously presented by the Pasteur Monument Committee of France to the American people of assisting in the erection of a suitable monument at Paris to the memory of the great investigator, and that the President is hereby authorized to appoint a committee to raise a fund, which shall be presented in the name of the Association.

---

*Resolved*, That this Association approves the action of the Master Horseshoers' Association in reference to the instruction of its members and their subordinates on such subjects as tend to perfect the art of horseshoeing, and thus avoid the great and frequent losses that result from the imperfect shoeing of the horse's hoof; and this Association pledges its support to all properly directed measures designed to accomplish this object; and it is the sentiment of this Association that horseshoers should be instructed in such subjects as pertain to the normal form and function of the foot and the mechanical processes necessary to preserve such conditions.

---

*Whereas*, Tuberculosis of some of our domestic animals, and especially of cattle, is a widespread and destructive disease; and,

*Whereas*, Statistics accumulated during the past year show that the disease is very prevalent throughout this country, especially in dairy herds, and indicate that it is steadily increasing, except in States where active measures for its suppression have been enforced; and,

*Whereas*, There exists in some quarters a difference of opinion as to the relation of tuberculosis among cattle to the public health, notwithstanding the fact that this matter has been the object of careful scientific inquiry by a great number of eminent scientists in all parts of the world, and that reliable and uniform results and observations are recorded in great numbers in the veterinary and medical literature; be it

*Resolved*, That it is the opinion of the United States Veterinary Medical Association that the following points have been demonstrated beyond dispute and may be accepted as fully established:

1. That tuberculosis of man and cattle are identical.

2. That the milk of cows with tuberculous udders may cause tuberculosis in animals fed upon it.

3. That the milk from cows with extensive tuberculosis but apparently healthy udders may in some cases contain the germs of tuberculosis and cause the disease in animals fed upon it.

4. That in some cases the germs of tuberculosis appear in the milk of tuberculous cows that are not far advanced in the disease, and that have udders that are healthy, so far as can be determined by an examination made during the life of the animal.

5. Slightly tuberculous cows sometimes succumb to a sudden exacerbation of tuberculosis and furnish virulent milk for a period before it is possible to discover their condition by means of a physical examination.

6. Tuberculin furnishes incomparably the best means of recognizing tuberculosis in the living animal.

7. Tuberculin, properly used for diagnostic purposes, is entirely harmless to healthy cattle, and is so exceedingly accurate in its effects that the few errors resulting from its use cannot affect the general results, and are of less frequent occurrence than follow the use of any other method of diagnosing internal diseases.

8. That the carcasses of tuberculous animals may be and sometimes are dangerous to the consumer, and all such carcasses should be subjected to rigid inspection by a competent veterinarian, and that those that are condemned should be disposed of in such a manner that it will be impossible to put them on the market for consumption as human food.

9. That the importance of dairy inspection cannot be overestimated, and municipal and health authorities should at once perfect a system commensurate with the vast importance of the subject.

*Resolved*, That the live-stock and especially the breeding interests of this country can never regain their former prosperity until such measures have been carried out by the National and State Governments as will afford some reasonable guarantee against the continued ravages of this disease. And in view of the prevalence of bovine tuberculosis in foreign countries, and the measures taken by some of them to protect their cattle from further infection, the United States should prohibit the importation of breeding animals until they have been proven by the tuberculin-test to be free from this disease.

